# An XML transfer schema for exchange of genomic and genetic mapping data: implementation as a web service in a Taverna workflow

**DOI:** 10.1186/1471-2105-10-252

**Published:** 2009-08-14

**Authors:** Trevor Paterson, Andy Law

**Affiliations:** 1 Division of Genetics and Genomics, The Roslin Institute and Royal (Dick) School of Veterinary Studies, The University of Edinburgh, Edinburgh, EH25 9PS, UK

## Abstract

**Background:**

Genomic analysis, particularly for less well-characterized organisms, is greatly assisted by performing comparative analyses between different types of genome maps and across species boundaries. Various providers publish a plethora of on-line resources collating genome mapping data from a multitude of species. Datasources range in scale and scope from small bespoke resources for particular organisms, through larger web-resources containing data from multiple species, to large-scale bioinformatics resources providing access to data derived from genome projects for model and non-model organisms. The heterogeneity of information held in these resources reflects both the technologies used to generate the data and the target users of each resource. Currently there is no common information exchange standard or protocol to enable access and integration of these disparate resources. Consequently data integration and comparison must be performed in an *ad hoc *manner.

**Results:**

We have developed a simple generic XML schema (GenomicMappingData.xsd – GMD) to allow export and exchange of mapping data in a common lightweight XML document format. This schema represents the various types of data objects commonly described across mapping datasources and provides a mechanism for recording relationships between data objects. The schema is sufficiently generic to allow representation of any map type (for example genetic linkage maps, radiation hybrid maps, sequence maps and physical maps). It also provides mechanisms for recording data provenance and for cross referencing external datasources (including for example ENSEMBL, PubMed and Genbank.). The schema is extensible via the inclusion of additional datatypes, which can be achieved by importing further schemas, e.g. a schema defining relationship types. We have built demonstration web services that export data from our ArkDB database according to the GMD schema, facilitating the integration of data retrieval into Taverna workflows.

**Conclusion:**

The data exchange standard we present here provides a useful generic format for transfer and integration of genomic and genetic mapping data. The extensibility of our schema allows for inclusion of additional data and provides a mechanism for typing mapping objects via third party standards. Web services retrieving GMD-compliant mapping data demonstrate that use of this exchange standard provides a practical mechanism for achieving data integration, by facilitating syntactically and semantically-controlled access to the data.

## Background

Data integration across disparate datasources is a common problem, not limited to the sphere of bioinformatics, and has been addressed in many ways [1,2]. Cross indexing and hypertext linking of resources has historically been used for accessing bioinformatics data (e.g. SRS [3]) but is curation intensive and does not adequately address different datatypes found in the distributed datasources. However, cross referencing is widely used and forms the basis of the highly successful Entrez data retrieval system [4]. A common approach for integrating bioinformatics data is to warehouse data according to a shared data schema (e.g. ENSEMBL Biomart [5]). Data in the individual data sources must be mapped to the shared schema, capturing both the syntax of the data structure and the semantics of what the data objects represent. The warehousing approach therefore incurs heavy data curation costs in creating and maintaining the warehouse and requires extensive computing resources; the alternative approach of federating the warehouse introduces further problems in distributing queries across the underlying distributed resources.

An alternative recent approach involves attempting to integrate data semantically through the use of defined ontologies describing a particular data domain. In addition to the widely used Gene Ontology [6] which provides a controlled vocabulary for annotating gene and gene function attributes that can then be used in index based retrieval systems, other formal ontologies are actively being developed to support bioinformatics, particularly under the OBO umbrella (Open Biological Ontologies [7]). Well-defined, detailed ontologies have been created representing common concepts used across the bioinformatics domain, e.g. for sequence features (the Sequence Ontology [8]) and common concept relationships (the OBO Relation Ontology [9]) and also more specific concepts for particular domains, e.g. the Amphibian Gross Anatomy [10]. These ontologies provide excellent controlled terminologies for data description and annotation, but do not provide data transport protocols and provide neither the structure nor semantics required for data integration. Consequently, whilst ontology based descriptions of data objects can be compared across data sets – two objects with similar annotations may share similar properties – these annotations do not capture or assert actual relationships between the data objects.

Technologies developed to support the 'Semantic Web' such as OWL, the 'Web Ontology Language' provide an approach for exposing and integrating semantically consistent datasources (i.e. where multiple resources are represented in a common formal ontology) and the potential for Semantic Web integration of bioinformatics resources is being actively explored (e.g. Yeasthub [11]). Recently we have been involved in the ComparaGRID project which aims to integrate genomic data by mapping data sources to a shared OWL ontology for genomic information, thus facilitating semantic integration and query [12]. However, such an approach is computationally expensive and as yet difficult to implement using available semantic ontology tools.

A more lightweight and arguably more practical approach for data exchange is to encourage the curators of data resources to provide data export facilities in a common simple exchange format, which both captures the data structure and provides a degree of semantic clarity to the exported data, without providing the exacting constraints of a formal ontology. XML has long been recognized as a useful data structure with which to implement formal exchange formats [13]. Such an approach allows application developers to develop their integration systems against the common data format, where the structure of the data is unambiguous, and the semantics are sufficiently defined for usability.

Here we present our approach of using a simple structured XML schema (XSD) to define the data exchange structure for genetic mapping information, an approach successfully used for integrating data in other bioinformatics domains (e.g. Taxonomy [14]) and also for specifying standards defining the key information to include when reporting experimental results (e.g. those hosted at MIBBI [15]). Use of common exchange schemas to define data structures allows users (including consuming services or applications) to parse the data syntax into semantically described data objects. Our Genomic Mapping Data (GMD) schema allows common information types for our domain (and the relationships between these objects) to be represented in a syntactically defined structure, thus enabling GMD conformant data documents to be programatically parsed into meaningful genetic information. Furthermore, the GMD schema is extendable to handle additional datatypes. Extension may be achieved not only by evolution of the schema, but simply by using datatypes defined elsewhere, for example by importing additional schemas to the data document, or referring to controlled vocabularies or external ontologies.

To illustrate how the schema can be used we have built web services which export data from the ArkDB genomic mapping resource using our exchange format. We also illustrate how additional defined terms can be imported from a second schema which defines common data relationships relevant to the genomic mapping domain. We demonstrate how the Taverna Workbench can consume these web services into workflows to harvest and use mapping data for bioinformatics tasks. This is possible because Taverna can parse the XML data structure of the data documents provided as web service responses in the context of the defined data format (referenced as the GMD XSD schema).

## Results and discussion

### Schema Design

One approach for developing a shared schema for data exchange in a given information domain is to initiate a consultation and prototyping cycle amongst interested parties (data providers and users). However, this 'design by committee' approach is frequently protracted and contentious. In order to 'kick start' development of a usable standard for genomic mapping data we have developed a candidate exchange schema that seeks to capture the important core data concepts and relationships, whilst being potentially expandable to incorporate additional requirements if necessary for wider use. To encourage adoption, a generic, unambiguous and semantically 'neutral' schema is desirable. Indeed many of the common data objects in the genomic mapping domain cannot be defined unambiguously across user groups (for example the concepts of 'Gene', 'Map', and 'Marker'). Therefore the schema avoids precise semantic definition of concepts and leaves interpretation to individual data providers and applications. However, the structured syntax of the schema allows any important 'relationships' between concepts to be fully represented. Furthermore, because XML data documents can reference multiple schemas, users can further define their information by reference to additional external schemas. The generic nature of our mapping schema allows any type of map to be represented, allowing integration of the many types of data found in genetic resources. For example the representation of genetic linkage maps and sequence maps is conceptually and syntactically the same: the positioning of mapped objects on a map object using a coordinate system.

The prototype schema GenomicMappingData.xsd, (GMD: provided as additional file 1: GenomicMappingData.xsd and with further documentation at [16]), includes in-line descriptive annotations. Example data exchange documents conforming to the schema are provided as additional files 2 and 3 (demo1result1.xml, demo2result.xml), and afford examples of the data structures decribed below. We are also hosting an interactive WIKI site [17] to provide further guidance on best practice for schema usage, and as a forum for discussion of schema refinement and evolution by interested data providers and users.

The structure of our GMD schema aims to be clear and logical, but is primarily designed for unambiguous data storage and programmatic parsing rather than 'human readability'. The root Element of the document is <DataSet>, which is itself defined as a 'complex type', thus allowing this 'type' (gmd:DataSet) to be referenced in other XML documents that import the GMD schema. For example, this allows a web service description document (WSDL) to specify a return type of 'gmd:DataSet' for a particular web service, thus enabling parsing of returned result documents by a web service client (such as a Taverna web service workflow) according to the defined schema structure (see below).

The children of the <DataSet> root Element are multiple, single-copy containers for the data objects (e.g. <SpeciesContainer>, <Chromosomes>, <Markers>) and for binary data relationships(<Relationships>), assertions about data (<Assertions>), third-party datasources such as PubMed or ENSEMBL(<DataSources>), references to these (<ExternalReferences>) and a <Metadata> container for information about the provenance of the DataSet document.

All the main data Elements in the schema have an 'id' Attribute, which might typically be the source database ID. An 'id' Attribute should be unique within a document, but this is not enforced in the schema. These data objects represented by Elements with an 'id' Attribute can be reused by 'foreign key' reference in the document by various 'reference type' Elements. These have an 'idref' Attribute, which should reference an extant 'id' Attribute of another Element (again this is not schema-enforced to prevent programmatic parsing from failing due to validation errors caused by minor inconsistencies in a DataSet). For example, a <Species> Element with 'id = x', can be referenced by a <SpeciesREF> Element with 'idref = x', in this case efficiently allowing multiple objects to declare that they pertain to a particular organism. Similarly, various mapping objects may reference a particular <Analysis> (experimental set) through an <AnalysisREF>, providing important contextual information. In addition, the top level containers are allowed document unique 'id' Attributes of type 'xsd:ID'. In practice, this greatly assists programmatic parsing of the document as such Elements can be obtained from a Document Model by their 'id' value.

The information about each data object is held in subsidiary Elements. In designing the schema, Elements were preferred over Attributes for data storage in order to allow the built-in Taverna parser ('XMLSplitter') to access data values. In order not to overly constrain schema users, most data Elements are 'optional' apart from where required for structural association. For example, a <Chromosome> Element optionally contains <ChromosomeName>, <DBIdentifier>, <DataAccessMethod>, <SpeciesREF> and <ExternalReferenceREFS> whilst a <Relationship> Element logically *must *contain <ObjectREF>, <SubjectREF> and <RelationshipType> Elements (see below). 'Terminal' data Elements generally have content of type 'xsd:string' or 'xsd:double', whilst those of REFType contain only an 'idref' Attribute (see above).

Several Elements such as <Units>, <QTLDetails>, <OtherData> and the <'Object'Type> child Elements (discussed further below) are allowed content defined as being of 'xsd:anyType', allowing any data structure (e.g. a simple string, an *ad hoc *XML data structure or an external datatype included by import from an external schema. This allows, for example, <Units> to be recorded as a free text string (e.g. "base pair", "centiMorgans", "flpter"), or perhaps as a type from the OBO Units of Measurement Ontology [18], by using the OWL Class URL (e.g.  for 'base pair'). This external type could be represented by a single Element of type xsd:string or xsd:anyURI, a type structure defined or not by schema import, or might be of gmd:REFType and point at an ExternalReference Element in the document itself (see above). Allowing <Units> to be defined as any type permits a generic representation of any map type data in schema-compliant DataSet documents. Of course inclusion of xsd:anyType extensions only allows defined client parsing if the extended types are referenced by import of an external XSD schema.

Structural nesting of Elements is used where logical, for example the <Mappings> that lie on a given <Map> are nested under that <Map>, and a sequence associated with a particular <Marker> or <Clone> may be nested as a <SequenceREF> reference under that object. However, any data relationship may alternatively be stored as a binary relationship (see below). Rather than provide multiple subsumption-typed versions of data objects such as Map, Sequence or Marker, which would cause gross schema inflation when accounting for all users' requirements, such Elements contain optional child Elements to record a specific 'ObjectType' for the object (such as <MapType>, or <SequenceType> for example) and are also allowed content of 'xsd:anyType', which, as described above, could hold a string value such as "Linkage Map", "Radiation Hybrid Map", "EST", "genomic". In order to promote data consistency within a user group, permitted string values might derive from enumerated (but expandable) lists, or from more controlled defined vocabularies, or indeed types could be imported from external sources (as above). This semantically 'loose' mechanism enables a 'Marker' object to be interpreted generically as an 'undefined genetic marker' or, as a specific type of marker (*e.g. *a "SNP", a "Gene") if such ObjectTypes are provided and documented by the data provider and are relevant to the consumer application.

An important feature of the DataSet schema is the provision of full data provenance and data access protocols; only by providing this information can reliable data integration be achieved. To this end an optional Element <DBIdentifier> is allowed for most data Elements which can be used to record the identifier or accession number for that object in the datasource (as recorded in the DataSet Metadata). In addition, the optional <DataAccessMethod> Element can detail how to actually access a record of this object from the source datatabase (as a service URL or by a parameterized Web Service call or other means). Providing this information enables programmatic expansion of datasets, for example where one dataset provides details of markers found on a particular map, and each marker provides its access method, a client program could fetch the full record for each marker, which might detail every map that this marker is associated with, its relationships to other markers or sequences and any external datasource links. In order to facilitate integration across datasources most data Elements in the schema may include external references to third party data repositories. This is provided by <External Reference> Elements which detail an external datasource name/location and a specific object ID therein. These are referenced internally in the document by optional <ExternalReferenceREF> child Elements for a given data Element, which point to a <External Reference> Element. For example this could allow a Sequence in the DataSet to be explicitly linked to a Sequence in GenBank by its GenBank accession. The client can then use this GenBank ID to retrieve the original record for this Sequence. This allows integrative workflows to be composed, for example using the Taverna Workbench (see below).

### Representing Data Relationships

A part of the knowledge domain that is typically hard to capture is represented by <Relationship> Elements, which capture (potentially directional) relationships between two data objects (captured as a <SubjectREF> and an <ObjectREF>, which reference a pair of data Elements in the document). In order to capture the nature of the Relationship each <Relationship> must be 'typed' via a <RelationshipType> child Element (again allowed content of 'xsd:anyType', as described above). Ultimately, use and interpretation of relationships will be dependant on a shared interpretation by data users and providers. Again data interpretation and consistency might be assisted by provision of a controlled 'vocabulary' of possible types (*e.g. *"homologous to", "orthologous to", "associated with", "derived from") or defined terms or types could be imported from external sources (e.g. the OBO Relation Ontology [9] or an extension thereof with relationships of greater relevance to our genome mapping domain). However, precise semantic definition of relationship types across the whole potential user domain is perhaps overly ambitious, and controlled vocabularies for particular sub-domains or 'user groups' may prove more pragmatic.

**Figure 1:**
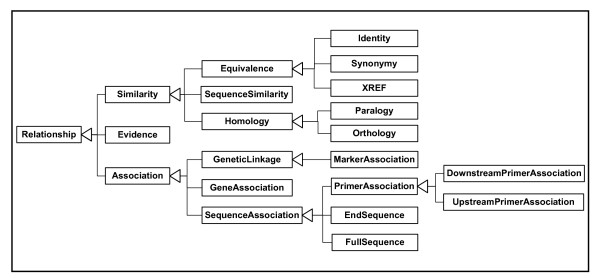
**The Hierarchy of Relationship Types in GenomicDataRelationships.xsd**. The GenomicDataRelationships schema (gdr, see additional file 4: GenomicDataRelationships.xsd) specifies an inheritance of xsd:complexTypes rooted on 'RelationshipType'. Textual definitions of these Relationships are provided by inline xsd:annotaions. A global Element is defined for each Type, with the document structure of the root Element 'Relationship' reflecting the inheritance pattern of the Types, which is shown here. Thus a 'SequenceSimilarity' is a type of 'Similarity' is a type of 'Relationship'.

By way of demonstration, we have defined a relationship type schema, GenomicDataRelationship.xsd, (gdr: provided as additional file 4: GenomicDataRelationships.xsd, and available with documentation at [19]). This schema defines complex types that represent real data relationship types in genomic datasets (based on the ArkDB database and others). The datatypes are derived by extension of parent types to represent the inheritance/subsumption hierarchy of relationship types. In order to visualize the relationship type inheritance in gdr, a global Element is declared for each type, and the schema places these Elements in a hierarchy mirroring the inheritance pattern of the types (see Figure 1). These types can be included in a GMD:DataSet document by import as types for <RelationshipType> and <SequenceRelationship> Elements (e.g. <RelationshipType xsi:type="gdr:OrthologyType">) or by adding content Elements (e.g. <RelationshipType><gdr:Orthology/></RelationshipType>).

An important feature of <Relationship> Elements, in common with other data Elements, is that they can record the provenance of the assertion through <ExternalReference> Elements, allowing users to validate and accept or reject these relationships.

### ArkDB web services

Roslin Institute curates an integrated genomic mapping database for farm animal genetics, ArkDB [20]. Mapping data can be accessed by an open access web application with data being displayed textually or explored graphically as aligned maps. However, users would benefit from the provision of bulk data export facilities to allow reuse of the mapping data for their own analyses and applications, for example integrating ArkDB data with their own data, and potentially with data from third-party data sources. The semantic complexity of the genomic data held in ArkDB is beyond that of existing genomic exchange formats, typically delimited flat-file formats which have been designed primarily to accommodate sequence annotation information (e.g. GFF [21]) or ad hoc lightweight data serialisation formats such as YAML [22]. In order to accommodate the full diversity of information held in ArkDB we have built a suite of web services (Table 1) which return mapping data structured as GMD validating documents. Services are built and deployed on the XFire platform [23], with auto generation of service WSDLs. The services are exposed singly and as an aggregated WSDL service. This aggregated WSDL has been modified to import the GMD schema and to specify that all of the services return data of type gmd:DataSet (*i.e. *an XML document conformant (and validating) with the GenomicMappingData.xsd schema).

**Table 1:** ArkDB Web Services

**Service**	**Method**	**Parameters**
ArkObjectService	fetchArkObject	objectAccession

SpeciesService	fetchAllSpecies	null

KaryotypeService	fetchSpeciesByAccession	speciesAccession

KaryotypeService	fetchNamedSpeciesKaryotype	speciesName

KaryotypeService	fetchAllSpeciesKaryotypes	null

KaryotypeService	fetchChromosomeForMap	mapAccession

MapService	fetchMapsForChromosome	speciesName chromosomeName

MapService	fetchMapsForChromosomeAccession	chromosomeAccession

MapService	fetchMapsForAnalysis	analysisAccession

MapService	fetchAllMapsForSpecies	speciesName

MapService	fetchUncontainedMapsForSpecies	speciesName

MapService	fetchUnassignedMapsForSpecies	speciesName

MappingAnalysesService	fetchAnalysesForSpecies	speciesName

MappingAnalysesService	fetchAnalysesForMap	mapAccession

MarkerService	fetchMarkerByAccession	markerAccession

MappingService	fetchMappingsForMap	mapAccession

ArkWebService	Single access point for all Methods	

Services and WSDLs are exposed at  (e.g. ). The WSDL at  uses the same services but specifies that returned documents conform to the GMD schema.

Individual services take a number of query parameters (as defined in the WSDLs, as named strings wrapped in XML) such as: a species or chromosome name or a database identifier. and return the response information as a DataSet document.

The services are implemented using the ArkDB web application's Java Model and API, and serialize Ark Objects to XML. Each individual service returns an internally consistent document filled in with appropriate data Element values for that query. For example, the SpeciesService provides a single *getAllSpecies *method that returns a document with limited details of all the Species represented in the ArkDB, *i.e. *only the Metadata and SpeciesContainer Elements contain data. On the other hand, the KaryotypeService provides a method to *getAllSpeciesKaryotypes *which additionally fills in Chromosome data in the Chromosomes container, where each Chromosome cross references the source Species. Further methods allow karyotypes to be retrieved by species name or ArkDB accession number. A more complicated service such as MapService provides more extensive information to the returned GMD document. For example, the *getMapsForChromosome *takes speciesName and chromosomeName parameters, and return data about Species, Chromosomes, Maps and Analyses. The actual Mappings on a Map are returned by a method in the MappingsService, which returns Mappings data as well as data about the mapped Markers, Clones, Sequences or other objects. These returned XML documents necessarily expand in size, but the XFire SOAP API efficiently handles data compression and transfer.

The service designer controls the level of detail provided in each request, with the ability to 'drill down' provided by chaining data parsed from the results of one request as input parameters to the next. For example, full details on an individual marker discovered on a MappingService request can be obtained by querying the MarkerService which will return not only mapping data about that marker, but also any Relationships held about that marker in the database, which for example might include known associations with other markers, genes or sequences and cross references to third party data resources.

### Taverna workflows using the ArkDB web services

The structure of the ArkDB web services described above allows requests to be chained together in a workflow to drill down and query through the data held in the database. Both the GMD schema and the web services were designed to be compatible with the Taverna Workbench application [24]. Taverna is an open-source workflow tool that allows users to construct analysis workflows from components located on both local and remote machines, and run these workflows incorporating their own data or query parameters. We have used methods from the ArkDB web services as external components in workflows and used the Taverna 'XMLSplitter' processor and java XPath 'widget' to parse through the structure of the returned documents to extract the data fields desired. In this manner it is possible to chain web services together, by extracting the required value(s) from one web service response and using it as the input of a subsequent query.

**Figure 2:**
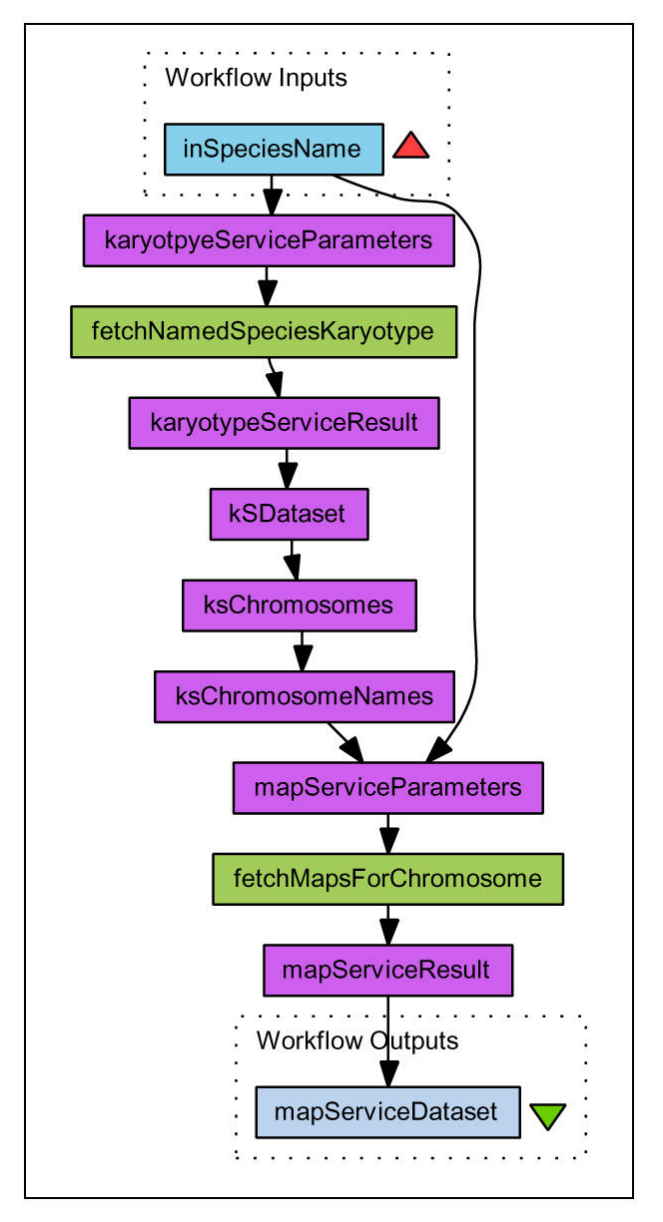
**Taverna Workflow 1**. Graphical representation of a workflow using ArkDB web services captured from Taverna Workbench v1.7.0.0. A species name is handed in as a parameter to karyotypeService. The result returned from this web service is a DataSet document conforming to the GenomicMapping Data schema, which can be parsed by the Taverna XMLSplitter, to drill down through Elements in the document structure to retrieve the chromosome names for the input species name parameter. Together with the initial species name each chromosome name in turn is entered as a parameter for iteration over the fetchMapsForChromosome service, which returns a set of DataSet documents each containing the list of available maps for each specified chromosome. The Workflow document is available as additional file 5: demoWorkFlow1.xml, and one of the resulting data documents is available as additional file 2: demo1result1.xml.

In an example workflow we join two service methods '*fetchNamedSpeciesKaryotype*' and '*fetchMapsForChromosome*' by extracting all the Chromosome Names from the first response and chaining into an iteration over the second service, passing in the parsed Chromosome Name from the first result set together with original Species Name. The workflow is shown in Figure 2 and the Workflow document is available as additional file 5: demoWorkFlow1.xml. When initiated with the input parameter Species Name 'Pig' the resulting output is a set of 20 DataSet documents which are conformant with the GMD schema, each containing details of the maps found on one of the chromosomes. (One such result document is available as additional file 2: demo1result1.xml.) The level of detail for each map is determined by the '*fetchMapsForChromosome*' service and includes Species, Chromosome, Map, and Analysis data. The details of genetic entities mapped on these maps could be obtained by parsing out each Map Accession, again using the Taverna XML Splitter, and passing this as the input mapAccession parameter for the '*fetchMappingsForMap*' service. Additional Taverna Processors could be added as widgets to the workflow that could generically parse any result document, because they all conform to the same XSD schema. For example it would be trivial to add an XSLT processor or Java DOM processing bean to combine multiple result sets into a single DataSet document.

**Figure 3:**
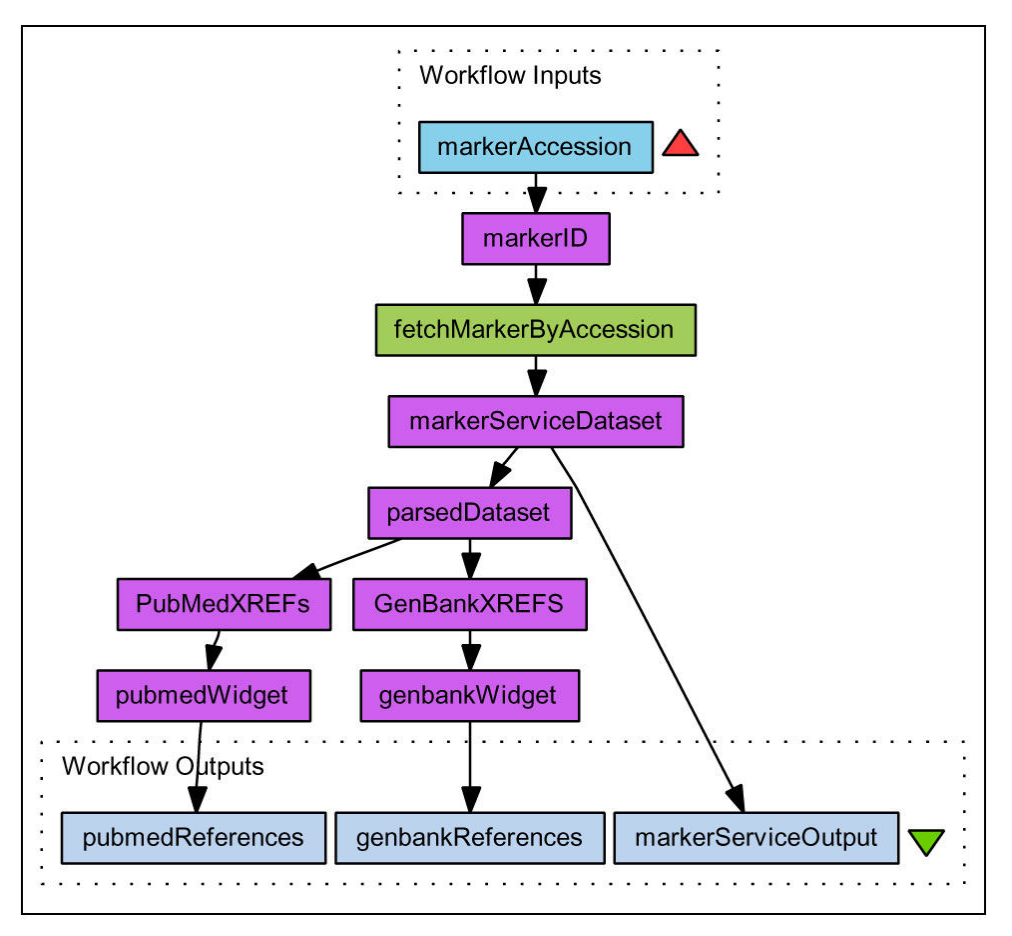
**Taverna Workflow 2**. Graphical representation of a workflow using ArkDB, PubMed and GenBank web services captured from Taverna Workbench v1.7.0.0. An ArkDB accession number for a Marker (which could itself be retrieved from another ArkDB web service) is handed in as a parameter to the ArkDB fetchMarkerByAccession service. The result returned from this web service is again a DataSet document conforming to the GenomicMappingData schema, which can be parsed by the Taverna XMLSplitter, to retrieve the DataSet Element and the ExternalReferences Element, which is then parsed by the Taverna 'XPath from Text' java widget to retrieve the SourceIDs for any PubMed and GenBank external references (xrefs) in this result document. (An XPath expression to retrieve all GenBank IDs from the document being '//gmd:ExternalReference/gmd:DataSource/@idref [.="GenBank"]/../../gmd:SourceID'). These IDs are used to parameterize requests to PubMed and GenBank web services to retrieve the source records for these xrefs, using Taverna NCBI java widgets ('Get PubMed XML by PMID' and 'Get Nucleotide GBSeq XML'). In this case the Workflow outputs the original request document which details all of the information held in ArkDB about this Marker (its Mappings, and any Relationships to other objects such as Markers and Sequences) and also documents for any referenced PubMed citations or GenBank sequences referred to in the result document. The Workflow document is available as additional file 6: demoWorkFlow2.xml, and the resulting data document is available as additional file 3: demo2result.xml.

The use of additional widgets to join third party datasources is demonstrated in Figure 3. The Workflow document is available as additional file 6: demoWorkFlow2.xml, and the resulting data document is available as additional file 3: demo2result.xml. In this workflow, details of a single marker are retrieved and any external references to PubMed or GenBank Sequence resources are parsed using the Taverna XPath widget. The XML records for these are then retrieved from NCBI using Taverna widgets that connect to NCBI web services. The results of running this workflow with the input *markerAccession *= 'ARKMKR00023953' include two Pubmed documents, a Genbank Sequence document and the details of mappings of this marker on four maps in ArkDB. Because Taverna is able to 'scavenge' (import) publicly available web services, in particular any service described by a WSDL document, workflows can access and therefore integrate data held in a wide range of bioinformatics resources. If further genomic mapping resources provided WSDL accessible data – formatted according to our proposed schema – then semantically meaningful data integration across resource and species boundaries would be possible using the tools currently available in Taverna.

**Figure 4:**
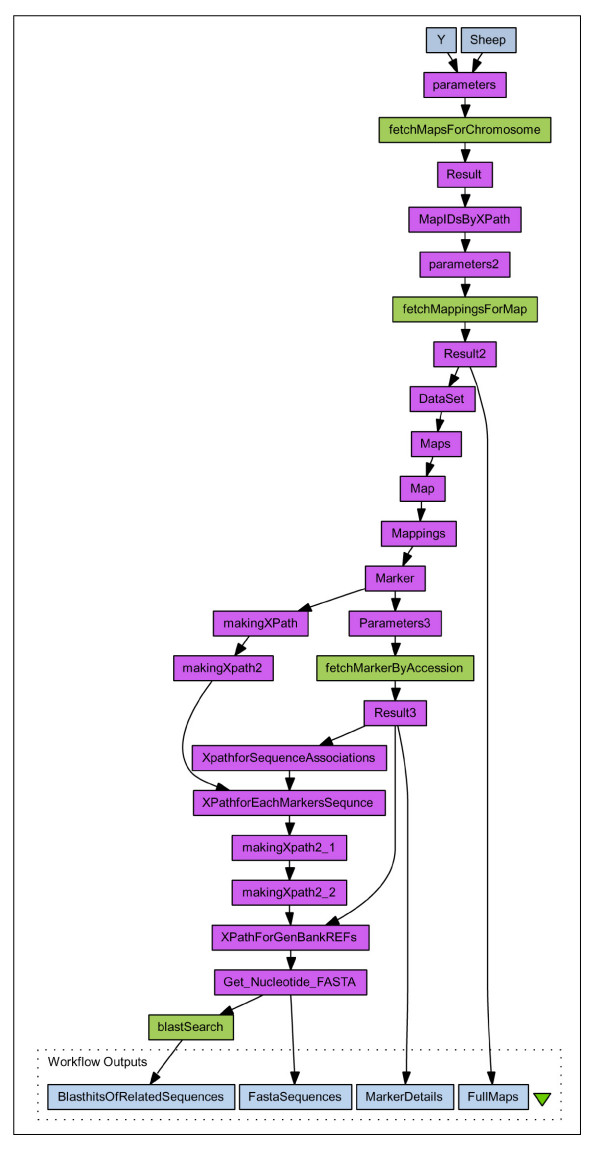
**Taverna Workflow 3**. Summary representation of a Workflow that uses ArkDB, DDBJ and GenBank web services to find potential sequence homologies to Sheep Chromosome Y, captured from Taverna Workbench v1.7.0.0. (The actual Workflow is more detailed and uses a temporary file to reduce the number of calls to the external services by removing GenBank sequence ID redundancy, this diagram is available as additional file 7: fig5.pdf, and the Workflow document as additional files 8, 9 and 10: demoWorkFlow3.xml, appendtoFile.xml, listFromFile.xml). The input parameters 'Sheep' and 'Y' are used to retrieve all Maps for this Chromosome from ArkDB, the Map accession numbers are used to retrieve full details of all these Maps including all the Mappings of Markers, and the Marker accession numbers are used to retrieve full details of all the Markers on these Maps. Where these Markers are associated with a sequence, the fasta formatted sequences are retrieved from GenBank and these are used to perform a BlastN search using DDBJ services. All GMD DataSet documents are output for the Map and Marker details, as are the fasta sequences from GenBank and the Sequence similarities found at DDBJ.

Figure 4 demonstrates a more useful workflow created in Taverna, which is capable of finding sequence similarities to any chromosome represented in ArkDB. (The actual workflow is more detailed and uses a temporary file to reduce the number of calls to the external services by removing GenBank sequence ID redundancy, this diagram is available as additional file 7: fig5.pdf, and the Workflow document as additional files 8, 9 and 10: demoWorkFlow3.xml, appendtoFile.xml, listFromFile.xml). In this case the full details for all Maps of a named Species Chromosome are retrieved, together with full details of all the Markers, and any referenced Sequences associated with these markers are fetched from GenBank. These sequences are then used to perform a BlastN search against a sequence database (here we use a simple blast service provided by DDBJ [25]) which will potentially find any cross species homologies to markers mapped to the input chromosome. When the workflow is run with the input parameters 'Sheep' and 'Y', 5 maps are found in ArkDB with a small number of Markers on each. Four of these are associated with sequences retrieved from GenBank, and BlastN comparisons are then performed upon these. Further additions to the workflow could be used to parse out the details of sequences of interest to retrieve possible links to homologous and potentially orthologous regions of chromosomes of other species. Many web services can return XML formatted documents, with associated XSD schemas, which facilitates this parsing and chaining of services (e.g. EBI's SOAP services [26]).

## Conclusion

We have developed a data exchange standard for genomic mapping data that allows us to define the structure and general semantics of data exported via web services from our in house genomic mapping resources. Data exported in our data exchange standard can then be meaningfully integrated into workflows created using the Taverna Workbench. Adoption of this exchange format (or an evolution of it) by further data providers will allow integration of mapping data from distributed data resources, either by incorporation of WSDL described web services into Taverna workflows, or by any bespoke web service client application.

Our exchange format provides several important features to facilitate its generic use by a wide community of data providers and consumers. (1) We have aimed for a relaxed semantic definition of the data objects found in the mapping world. This should simplify the data mapping process, both that performed by the data provider (from data source to schema) and between data resources when data users integrate data from multiple sources. (2) We provide extensibility within the schema, both to allow additional information to be included in GMD conformant documents, and to include specific DataTypes from third party sources such as ontologies or other data schemas. (3) We provide an External Reference mechanism to carry references to third party data resources; obvious uses for this are for PubMed citations, Genbank accessions or ENSEMBL gene IDs. (4) We provide a structure to record any type of relationship between two data objects, thus not constraining the document format to the common mapping relationships with which we are familiar. (5) We provide mechanisms to record the provenance of data: by detailing the datasource (as Metadata for the whole document, and by recording source identifiers and access methods for individual data Elements) and by allowing reference to external datasources both for individual data Elements and any for individual data relationships and assertions. (6) We provide a mechanism for recording *ad hoc *assertions about data objects which should allow flexibility in representing nuanced observations about the data, for example third party comments that a particular piece of data is obsolete or incorrect.

## Authors' contributions

AL conceived of the study, and participated in its design and coordination. TP designed the data transfer schema, implemented the ArkDB webservices, the demonstration workflows and drafted the manuscript. Both authors read and approved the final manuscript

## Supplementary Material

Additional file 1 **GenomicMappingData.xsd**. Proposed GenomicMappingData XML schema (XSD) for exchange of genomic mapping data. Click here for file

Additional file 2 **demo1result1.xml**. One of 20 GMD conformant data documents generated by executing demoWorkFlow1 in Taverna. Click here for file

Additional file 3 **demo2result.xml**. The GMD conformant data document generated by executing demoWorkFlow2 in Taverna (this workflow also returns XML documents from Pubmed and Genbank web services). Click here for file

Additional file 4 **GenomicDataRelationships.xsd**. Example XML schema (XSD) for defining relationships between genomic mapping data. Click here for file

Additional file 5 **demoWorkFlow1.xml**. Example Taverna Workflow (shown in Figure 2). Click here for file

Additional file 6 **demoWorkFlow2.xml**. Example Taverna Workflow (shown in Figure 3). Click here for file

Additional file 7 **fig5.pdf**. Full graphical representation of the Taverna Workflow summarized in Figure 4. Click here for file

Additional file 8 **demoWorkFlow3.xml**. Example Taverna Workflow (shown in Figures 4 and 5). Click here for file

Additional file 9 **appendtoFile.xml**. Nested Taverna Workflow referenced in demoWorkFlow3.xml (place in root directory). Click here for file

Additional file 10 **listFromFile.xml**. Nested Taverna Workflow referenced in demoWorkFlow3.xml (place in root directory). Click here for file
