# Supplementary figures and images for: An XML transfer schema for exchange of genomic and genetic mapping data: implementation as a web service in a Taverna workflow
Source: BMC Bioinformatics. 2009 Aug 14;10:252. doi: 10.1186/1471-2105-10-252 (PMC2743669; doi:10.1186/1471-2105-10-252)

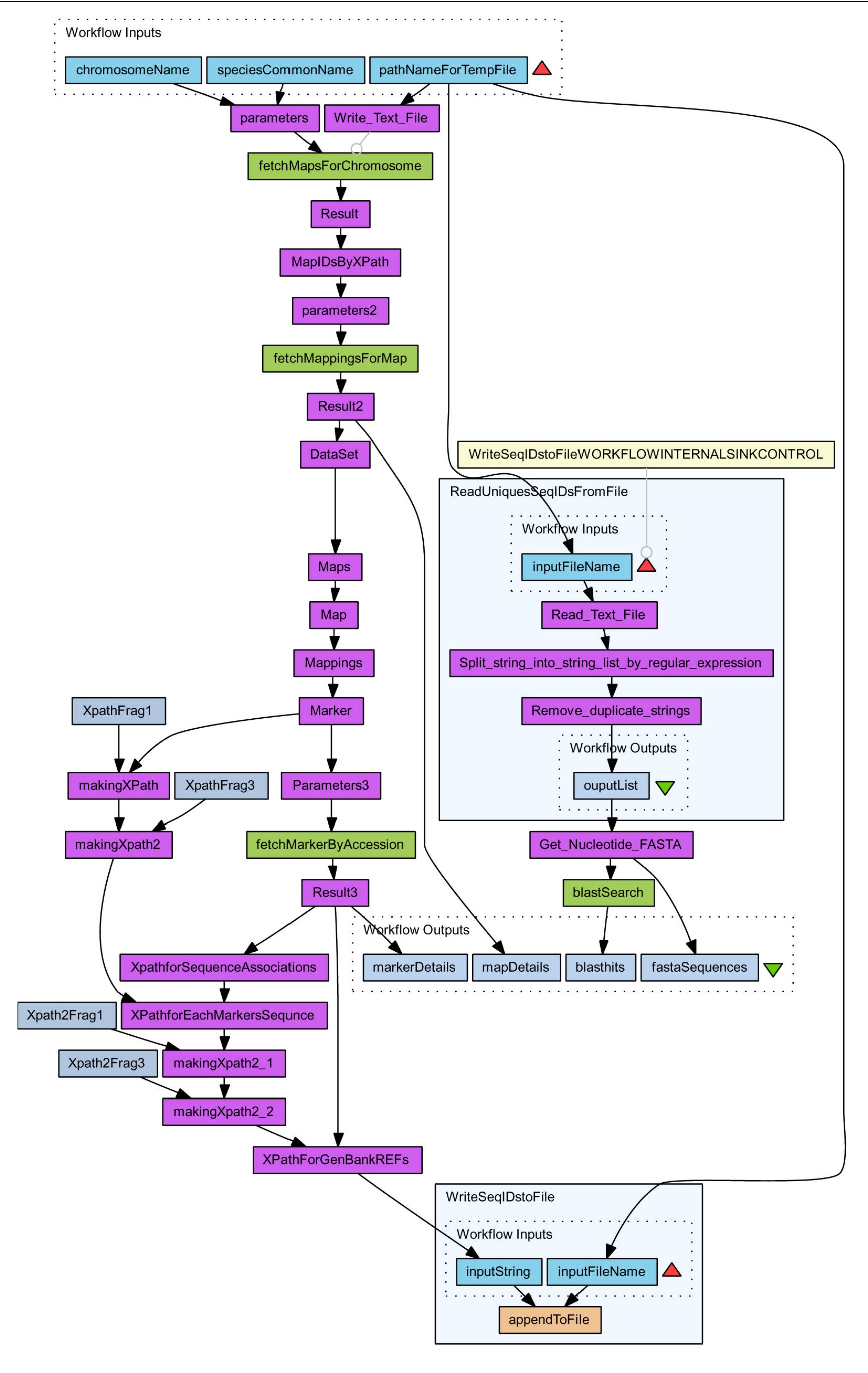

Supplement: Additional file 7 — fig5.pdf. Full graphical representation of the Taverna Workflow summarized in Figure 4. [file 1471-2105-10-252-S7.zip › sup7.pdf]
